# Highly Variable Recombinational Landscape Modulates Efficacy of Natural Selection in Birds

**DOI:** 10.1093/gbe/evu157

**Published:** 2014-07-24

**Authors:** Toni I. Gossmann, Anna W. Santure, Ben C. Sheldon, Jon Slate, Kai Zeng

**Affiliations:** ^1^Department of Animal and Plant Sciences, University of Sheffield, United Kingdom; ^2^School of Biological Sciences, University of Auckland, New Zealand; ^3^Edward Grey Institute, Department of Zoology, University of Oxford, United Kingdom

**Keywords:** protein evolution, natural selection, Hill–Robertson interference (HRI), tissue specificity in gene expression, recombination, RNAseq

## Abstract

Determining the rate of protein evolution and identifying the causes of its variation across the genome are powerful ways to understand forces that are important for genome evolution. By using a multitissue transcriptome data set from great tit (*Parus major*), we analyzed patterns of molecular evolution between two passerine birds, great tit and zebra finch (*Taeniopygia guttata*), using the chicken genome (*Gallus gallus*) as an outgroup. We investigated whether a special feature of avian genomes, the highly variable recombinational landscape, modulates the efficacy of natural selection through the effects of Hill–Robertson interference, which predicts that selection should be more effective in removing deleterious mutations and incorporating beneficial mutations in high-recombination regions than in low-recombination regions. In agreement with these predictions, genes located in low-recombination regions tend to have a high proportion of neutrally evolving sites and relaxed selective constraint on sites subject to purifying selection, whereas genes that show strong support for past episodes of positive selection appear disproportionally in high-recombination regions. There is also evidence that genes located in high-recombination regions tend to have higher gene expression specificity than those located in low-recombination regions. Furthermore, more compact genes (i.e., those with fewer/shorter introns or shorter proteins) evolve faster than less compact ones. In sum, our results demonstrate that transcriptome sequencing is a powerful method to answer fundamental questions about genome evolution in nonmodel organisms.

## Introduction

It is well known that the rate of protein evolution varies across the genome (Li 1997). Determining the causes of this variation is a powerful way to quantify the relative importance of natural selection and genetic drift and to identify factors that are important in shaping patterns of molecular evolution (Kimura 1983; Li 1997; Pál et al. 2006). When protein-coding DNA sequences are analyzed, the rate of protein evolution is often measured by the ratio $\text{ω}=d_{\text{n}}/d_{\text{s}}$, where *d*_n_ and *d*_s_ are, respectively, the rates of nonsynonymous and synonymous substitutions (Li 1997; Nei and Kumar 2000; Yang 2006). By using the ratio of *d*_n_ to *d*_s_, ω is expected to be less sensitive to variation in mutation (Nei and Gojobori 1986; Goldman and Yang 1994; Li 1997), which is known to exist in the genome (Lynch 2010; Hodgkinson and Eyre-Walker 2011). Therefore, variation in ω is considered to reflect selective pressures on the protein (Li 1997; Nei and Kumar 2000; Yang 2006). Specifically, under the assumption that synonymous changes are neutral, ω<1 is regarded as evidence of purifying selection acting on nonsynonymous mutations, ω=1 reflects neutral evolution, and ω>1 can be viewed as support for past episodes of positive selection driving nonsynonymous mutations to fixation (Li 1997; Nei and Kumar 2000; Yang 2006).

Estimates of ω have been obtained from a large array of different taxa. ω is generally less than 1 when considering the genome as a whole, reflecting the widely accepted theory that most nonsynonymous mutations have harmful effects on fitness and are therefore removed by purifying selection (Pál et al. 2006; Eyre-Walker and Keightley 2007). However, ω varies substantially across genes in the genome. Attempts to understand biological/selective causes of this variation have uncovered that ω is associated with factors such as protein dispensability, protein structure and stability, the number of protein–protein interactions, developmental timing, and patterns of gene expression (both in terms of expression level and tissue specificity); the extensive literature on these topics have been reviewed by Pál et al. (2006) and Choi and Hannenhalli (2013) (see also Marais et al. 2005; Parmley et al. 2007; Axelsson et al. 2008; Larracuente et al. 2008; Cai and Petrov 2010). Gene expression pattern appears to be a major correlate of protein evolutionary rate. For instance, in multicellular organisms, broadly expressed genes tend to have lower ω than genes with high tissue specificity in expression (Duret and Mouchiroud 2000; Axelsson et al. 2008; Larracuente et al. 2008; Slotte et al. 2011). Furthermore, genes involved in certain biological processes such as immunity and reproduction (e.g., spermatogenesis) tend to evolve faster than other genes in the genome and are often enriched for targets of positive selection, probably as a result of both inter- and intraspecies arms races (Nielsen et al. 2005; Haerty et al. 2007; Axelsson et al. 2008; Kosiol et al. 2008; Obbard et al. 2009). In contrast, genes with neural functions such as those expressed primarily in the brain exhibit lower evolutionary rates, which is likely to be a consequence of strong selective pressures to minimize the damaging effects induced by protein misfolding (Drummond and Wilke 2008). It should be noted that direction and intensity of correlations between ω and the factors mentioned above are sometimes inconsistent between species (reviewed by Pál et al. 2006 and Choi and Hannenhalli 2013), highlighting the importance of investigating these effects in distantly related species to verify their generality.

Variation in ω can also be induced by heterogeneity in recombination rate across the genome (Smukowski and Noor 2011). Physical linkage between loci on the same chromosome may affect the rate of protein evolution through Hill–Robertson interference (HRI), whereby any locus linked to other loci subject to directional selection experiences a reduction in local *N*_e_, the effective population size (Hill and Robertson 1966; McVean and Charlesworth 2000; Comeron et al. 2008; Sella et al. 2009; Charlesworth 2012; Cutter and Payseur 2013). Because the efficacy of selection is determined by $N_{\text{e}}s$, where *s* is the selection coefficient (Kimura 1983; Charlesworth B and Charlesworth D 2010), tight linkage between selected sites hinders both the fixation of beneficial mutations and the elimination of deleterious mutations. Recombination reduces the interference by breaking up the association between variants at different loci, which in turn increases *N*_e_, and hence the effectiveness of selection. The HRI theory therefore predicts that adaptive substitutions should appear more frequently in high-recombination regions, whereas low-recombination regions may accumulate more fixations of slightly deleterious mutations.

Despite having clear theoretical predictions, the importance of HRI in shaping protein evolution remains unclear (Webster and Hurst 2012; Cutter and Payseur 2013). In fact, as pointed out in a recent review, empirical studies have documented “extreme disparities among species” (Cutter and Payseur 2013). In *Drosophila melanogaster*, ω in regions that lack recombination (e.g., the fourth chromosome) is significantly higher than in regions where crossing-over occurs, consistent with relaxed purifying selection, but there is little difference in ω between regions with high, intermediate, and low crossover frequencies (Haddrill et al. 2007; Larracuente et al. 2008). In a recent analysis of the Drosophila Population Genomics Project data (Campos et al. 2014), it was found that the efficacy of natural selection, both positive and purifying, increases with local recombination rate, which may explain the lack of difference in ω within crossover regions, if the differential effects of positive and purifying selection on substitution rates at selected sites (the former elevates the rate and the latter depresses it) balance out (Campos et al. 2014). In contrast, in humans, recombination rate and ω were found to be uncorrelated, and there is little evidence that the efficiency of selection varies across regions with different recombination frequencies. This may be partly be explained by the small *N*_e_ in humans ($\approx 10^{4}$), which may render a general reduction in efficacy of selection, which in turn makes detecting the effects of HRI harder than in species with large *N*_e_ such as *Drosophila* ($\approx 10^{6}$; Bullaughey et al. 2008). As a third example, ω and recombination rate were found to be negatively correlated in yeast (e.g., Connallon and Knowles 2007; Cutter and Moses 2011). However, this pattern appears to be mediated by variation in gene expression, whereby slow-evolving, highly expressed genes tend to be located in high-recombination regions. After controlling for differences in expression, no evidence of substantial variation in selection efficacy across the yeast genome was found (Pál et al. 2001; Weber and Hurst 2009). These disparities between species call for analysis of data from species with different *N*_e_ and/or recombinational landscape, so that the HRI theory can be further tested and missing elements in the existing models identified (Webster and Hurst 2012; Cutter and Payseur 2013).

There are approximately 10,000 species in the class Aves (Jetz et al. 2012). Understanding how genome evolution occurs in this group of organisms has been an important topic in evolutionary genetics (reviewed by Ellegren 2013). In light of the discussion presented above, comparative genomic analysis of avian genomes will help to understand what factors, especially those characteristic of birds, correlate with ω, and whether these correlations are comparable to those observed in other species. Despite recent progress (reviewed by Ellegren 2013), important questions remain. For instance, it is unknown whether gene compactness (i.e., intron number, intron length, and protein length; reviewed by Choi and Hannenhalli 2013) is correlated with rates of protein evolution. It is also unclear whether genes situated in different genomic locations (e.g., subtelemeric versus central regions of macrochromosomes) tend to have different average specificity in gene expression. Answers to these questions are important for the study of HRI.

Avian genomes have a rather similar karyotype, with the number of chromosomes being almost constant across species (Griffin et al. 2007; Ellegren 2010; Skinner and Griffin 2012). A typical avian genome contains 40 pairs of chromosomes, of which, depending on definition, around a dozen are large macrochromosomes, and the remainder are microchromosomes. The lengths of macro- and microchromosomes can differ by more than an order of magnitude, which is substantially more than in, for example, mammals (reviewed by Ellegren 2010). Because at least one crossing-over per chromosome is needed for proper segregation during meiosis, a consequence of the large difference in chromosome size is that microchromosomes have substantially higher average recombination rates (≈10 cM/Mb) than macrochromosomes (0.5–2 cM/Mb), which has been confirmed by analyses of genetic maps in several birds (Stapley et al. 2008, 2010; Groenen et al. 2009; Backström et al. 2010; van Oers et al. 2014). This is more variable than average recombination rates observed in humans (1.07-2.10 cM/Mb; Jensen-Seaman et al. 2004). These genetic maps also reveal that, within macrochromosomes, the distribution of recombination frequency is nonuniform, with the majority of recombination events clustered in small regions close to telomeres. Although similar “telomere effects” have also been observed in other organisms such as humans (Jensen-Seaman et al. 2004), the clustering in birds appears to be stronger. For instance, the recombination rate drops very close to zero in regions more than 15 Mb away from the telomeres of zebra finch macrochromosomes (Backström et al. 2010; Stapley et al. 2010).

It has been suggested that HRI has probably played a role in driving the negative correlation between recombination rate (which is inversely related to chromosome size at a broad scale) and ω in birds (Axelsson et al. 2005; Nam et al. 2010; Künstner et al. 2010; Balakrishnan et al. 2013). However, the relative importance of positive and purifying selection to this observation is unknown. To provide better support for the HRI model, we intend to test 1) whether the elevation of ω in low-recombination regions is due to relaxed purifying selection, instead of enrichment of fast-evolving genes driven by positive selection and 2) whether positively selected genes are more likely to be found in high-recombination regions. In light of the highly variable recombinational landscape within macrochromosomes (Backström et al. 2010; Stapley et al. 2010; van Oers et al. 2014), it is essential to consider subtelomeric (i.e., ends) and central regions separately, which have high and low recombination frequencies, respectively.

Here, we focus on sequence divergence in protein-coding regions between two passerine birds, zebra finch and great tit (*Parus major*), with the latter being a model organism for addressing key topics in evolutionary ecology (Drent et al. 2003; Visser et al. 2003; Bouwhuis et al. 2010). By making use of a multitissue transcriptome data set in great tits (Santure et al. 2011) and the zebra finch genome (Warren et al. 2010), we seek to address the following questions: 1) How do variables such as tissue specificity in gene expression, intron number, intron length, and protein length correlate with ω? 2) do genes specifically expressed in different tissues evolve at different rates compared with other genes? and 3) is the efficacy of natural selection higher in regions with more frequent recombination, as predicted by the HRI theory?

## Materials and Methods

### Pairwise Sequence Alignments

The great tit transcriptome sequencing data were obtained from Santure et al. (2011). Briefly, in that article, normalized cDNA was sequenced from eight tissues; cDNA was pooled from ten different birds, all from Wytham Woods (Oxfordshire, UK). We focused on 95,979 assembled contigs with four or more reads. Because the contigs may contain noncoding sequences originating from pre-mRNA, UTRs, and other genomic parts, (e.g., due to leaky expression, Santure et al. 2011), we identified coding regions by mapping the contigs to cDNA of an outgroup species. We obtained outgroup information for *Gallus gallus* (chicken), *Taeniopygia guttata* (zebra finch), *Anas platyrhynchos* (mallard duck), *Ficedula albicollis* (collared flycatcher), *Meleagris gallopavo* (turkey), and *Melopsittacus undulatus* (budgerigar) from Ensembl (Flicek et al. 2012) and *Geospiza fortis* (medium ground finch) from Zhang et al. (2012). We used a nucleotide-based alignment strategy to map the great tit contigs to the corresponding regions of the outgroup genomes. First, we conducted a whole-genome BLAT (Kent 2002) search of the contigs against the cDNA of the outgroup species. For each pairwise BLAT hit, we obtained a pairwise alignment using bl2seq from BLASTALL (Altschul et al. 1990) and extracted from this alignment the longest ORF (minimum size 300 nucleotides) based on the outgroup sequence. The corresponding protein sequence of great tit was obtained by adjusting for frameshifts and stop codons, which were masked using PAL2NAL (Suyama et al. 2006). Input files for PAML (Yang 2007) were generated. We used rumode = −2 with the F3x4 codon model to obtain $d_{\text{n}}/d_{\text{s}}$ ratios using the codeml program of the PAML suite. Because one contig can have hits in multiple outgroup loci, we used the hit with the lowest *d*_s_ value. We also discarded hits for which the overall substitution rate was too high (tree length > 1.2, which is likely the effect of incorrect alignments). If one outgroup locus had several great tit hits, we combined the longest nonoverlapping stretches to obtain $d_{\text{n}}/d_{\text{s}}$ for this locus.

### Multiple Sequence Alignments and PAML Analysis

To conduct site-specific analyses of substitution rates, we used sequence triplets (3-way alignments) of chicken, zebra finch, and the great tit contigs. First, we identified homologous genes between chicken and zebra finch using Inparanoid (Remm et al. 2001; Ostlund et al. 2010). We then excluded those ortholog pairs, which either did not map or inconsistently mapped to the great tit contigs based on the pairwise sequence alignments. The remaining sequence triplets were aligned using MUSCLE (Edgar 2004). Uncertain sequence positions were removed based on scores from ZORRO (Wu et al. 2012), and the final alignment was processed with PAL2NAL (Suyama et al. 2006). The resulting alignments were used as input for PAML (Yang 2007). Because alignment errors may affect the downstream substitution rate analysis, to check the robustness of our results, we also applied a different alignment strategy using PRANK (Löytynoja and Goldman 2005) and GUIDANCE (Landan and Graur 2008; Penn et al. 2010). Only a small proportion of alignments were different between the two alignment strategies, and there was no difference in alignment inconsistencies between recombination jungles and deserts (G test, high vs. low/very low recombination rate and inner macrochromosomes vs. outer macrochromosomes and microchromosomes, *P* = 0.59 and *P* = 0.51), suggesting that alignment quality is only a minor issue in our case.

PAML uses a maximum likelihood approach to obtain substitution rate estimates for the provided phylogeny based on certain model assumptions. To obtain ω ($=d_{\text{n}}/d_{\text{s}}$) for each sequence triplet, we assumed a constant ω across the tree (model M0, one-ratio model). We also conducted site tests to identify heterogeneity in ω within genes (but not heterogeneity between branches). For this, the likelihood of a more complex model was compared with a nested simpler model, and significance was assessed using a likelihood ratio test. To test for evidence of positive selection, we compared the site models M7 and M8 (Yang et al. 1998, 2000). M7 and M8 assume that ω among sites follows a β(0,1) distribution, but M8 additionally allows for sites with ω>1. To identify genes with evidence for positive selection, we used a likelihood ratio test comparing M7 and M8 assuming a ${\text{χ}}^{2}$ distribution with df=2 and corrected for multiple testing using the method of Benjamini and Hochberg (1995) with false discovery rates (FDRs) ranging from 10% to 50%. Approximately 90% (depending on the applied FDR) of genes under positive selection based on the PRANK alignments are also detected when using MUSCLE alignments, suggesting that the majority of positively selected genes are consistent between the two alignment approaches. To test for the role of purifying selection for genes that did not show evidence of positive selection, we extracted parameter estimates from model M1a in PAML (Yang 2007), which allows a proportion of sites to be neutrally evolving (i.e., ω=1), and the remaining sites to be subject to purifying selection (i.e., ω<1).

### Physical Position and Estimates of Recombination Rate

Linkage maps constructed in several birds have consistently shown that 1) microchromosomes tend to have much higher per-site recombination rate than macrochromosomes and that 2) for macrochromosomes, most of the recombination events take place in subtelomeric regions with a large sections of the inner part of these chromosomes with much lower recombination rates (the telomere effect; Groenen et al. 2009; Backström et al. 2010; Stapley et al. 2010). We inferred the physical position of each gene using the zebra finch genome and classified genes into three categories: Microchromosome (chromosomes 13–28), macrochromosome with telomeric location (chromosomes 1–12 and Z within three megabases from the chromosome tip), and macrochromosome within the inner 25% of the total chromosome length. Our definition of microchromosomes followed that of Backström et al. (2010), which was based on the observation that recombination rates of chromosomes less than 20 Mb in length appear to be high (i.e., comparable to subtelomeric regions of macrochromosomes) and uniform across the length of the chromosome.

Even though the karotype within birds is relatively stable (Griffin et al. 2007), there were two major chromosomal fission and fusion events along the chicken and passerine lineages. We therefore excluded genes located at the beginning of chromosomes 1 and 4 as well as genes located at the end of chromosome 1A, where beginning and end are defined according to the zebra finch genetic map (Stapley et al. 2008). We also excluded genes on chromosome 4A, which is a microchromosome in the passerines but part of chromosome 4 (which is large) in chicken. We also classified genes according to the local recombination rates inferred by comparing the physical map with the genetic map in zebra finch (Backström et al. 2010). We defined three categories of genes according to their estimates of recombination rate as follows: Very low recombination (regions with no detected recombination events), low recombination (lower 25% of genes with nonzero recombination rate estimates), and high recombination (upper 75% of nonzero recombination rate genes).

### Extraction of Gene Features

We retrieved information on expression specificity for each gene from Santure et al. (2011) who followed the approach of Mank et al. (2008) to account for small levels of undetected expression. Expression specificity is measured by τ (Yanai et al. 2005), which ranges from 0 for genes with equal expression in all tissues to 1 for highly biased genes for whom most transcripts were found in only one tissue. Expression for each contig was standardized to the number of reads per million (TPM, see Santure et al. 2011 for details) and τ was calculated as follows;

$$\text{τ}=\frac{\sum_{i=1}^{N}\left(1-\frac{\text{ln}({\text{TPM}}_{i})}{\text{ln}({\text{TPM}}_{\text{max}}\text{)}}\right)}{N-1}$$

where *N* is the number of tissues, TPM*_i_* is the level of expression in tissue *i,* and TPM_max_ is the highest level of expression of a given contig over all *i* tissues examined. The number and size of introns, the size of exons, gene density (proportion of coding sites per Mb), and the chromosome size were inferred from the zebra finch genome (Warren et al. 2010). The proportion of sites near intron–exon boundaries was calculated as the number of introns divided by protein length.

### Statistical Analysis

The statistical package R was used to carry out statistical tests and generate box plots (using boxplot.stats function with default parameters). In a box plot, the box represents the range between upper and lower quartiles, the horizontal line within the box shows the median, and the whiskers show the most extreme data point, which is no more than 1.5 times the length of the box away from the box. To test for enrichment of genes with different gene ontology (GO) classifications, we used goatools (https://github.com/tanghaibao/goatools, last accessed July 28, 2014).

## Results

### Mapping the Great Tit Transcriptome to Other Avian Genomes

**Fig. 1.—:**
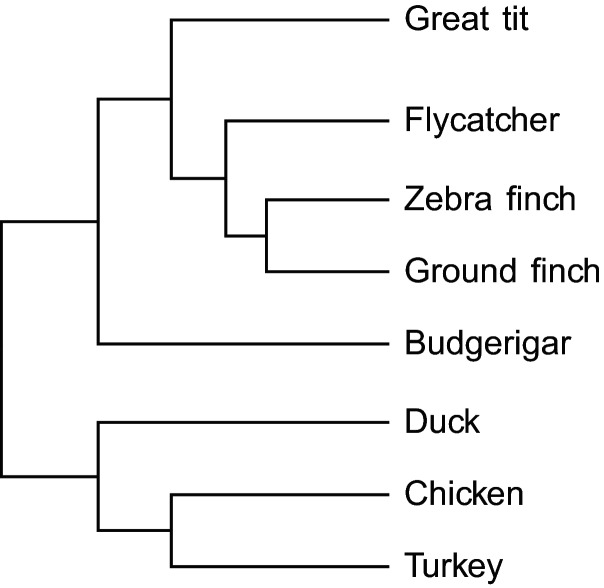
Phylogenetic relationship of great tit and seven bird species (Hackett et al. 2008).

We investigated rates of protein evolution by using a great tit (*P. major*) transcriptome data set based on RNA extracted from eight different tissues (brain, heart, kidney, liver, muscle, pancreas, skin, and testis/ovary; Santure et al. 2011). We mapped the contigs assembled by Santure et al. (2011) to the seven bird species for which a reference genome is available (see Materials and Methods). The median *d*_s_ between zebra finch and great tit is ≈0.1 (fig. 1 and table 1; see also supplementary fig. S6, Supplementary Material online), comparable to estimates reported earlier between zebra finch and other passerine birds (Künstner et al. 2010; Backström et al. 2013; Balakrishnan et al. 2013). The median *d*_s_ values obtained from pairwise comparisons between great tit and each of the four nonpasserine birds are also shown in figure 1 and table 1 (see also supplementary fig. S6, Supplementary Material online). The observed levels of synonymous divergence are consistent with the phylogenetic relationship of these species (Hackett et al. 2008, table 1 and fig. 1). For ω ($d_{\text{n}}/d_{\text{s}}$) between zebra finch and great tit, the median and mean are ≈0.1 and ≈0.16, respectively (supplementary table S1 and fig. S6, Supplementary Material online), which is again fairly close to the values reported for other passerine birds (0.08–0.13, Künstner et al. 2010; Backström et al. 2013; Balakrishnan et al. 2013).

**Table 1:** Median Estimates of *d*_s_ and $d_{\text{n}}/d_{\text{s}}$ Based on Pairwise Alignment between the Great Tit Transcriptome and Each of the Seven Different Bird Species (fig. 1)

Genome Reference	*d*_s_	$d_{\text{n}}/d_{\text{s}}$
Collared flycatcher (*Ficedula albicolis*)^a^	0.104	0.081
Zebra finch (*Taeniopygia guttata*)^a^	0.103	0.099
Medium ground finch (*Geospiza fortis*)^a^	0.111	0.083
Budgerigar (*Melopsittacus undulatus*)^b^	0.248	0.075
Mallard duck (*Anas platyrhynchos*)^b^	0.318	0.073
Chicken (*Gallus gallus*)^b^	0.339	0.074
Turkey (*Meleagris gallopavo*)^b^	0.346	0.071

^a^Passerine.

^b^Nonpasserine.

Because the quality of annotation is best for the zebra finch and chicken genomes, we used these two genomes as references. Specifically, we analyzed 8,294 two-way alignments between great tit and zebra finch; we were also able to obtain orthologous sequences from the chicken genome to construct three-way alignments for 5,460 genes. We focused on factors that may affect patterns of protein evolution between the two passerines, great tit and zebra finch.

### Correlates of Variation in Rates of Evolution in Passerines

We explored pairwise relationship between several genomic features and evolutionary rates in passerines (ω and *d*_s_) obtained from our two-way alignments, so as to identify predictors of evolutionary rates between the two passerines. Consistent with previous studies (Pál et al. 2006; Axelsson et al. 2008; Larracuente et al. 2008; Ekblom et al. 2010; Choi and Hannenhalli 2013), there is a highly significant positive correlation between ω and τ, a commonly used measure of tissue specificity (Yanai et al. 2005), which ranges from 0 (equal expression in all tissues) to 1 (highly biased expression with most transcripts found in only one tissue) (table 2). Because both τ and ω are significantly correlated with GC3 (GC content at 3rd codon positions), it is possible that the relationship between ω and τ is simply a by-product of these correlations. However, a partial correlation analysis suggests that this is not the case and that ω and τ are significantly positively correlated after variation in GC3 was controlled for (table 3, Case 1). To further rule out the possibility that the pattern is driven by a small number of genes with very high sequencing coverage (which may therefore have more accurate estimates of τ and potentially fewer assembling/sequencing errors), we introduced read depth as a second covariate and found that the positive correlation remains significant (table 3, Case 2).

**Table 2:** Pairwise Correlation Coefficients (Spearman’s ρ) for Variables Covary with the Rates of Protein Evolution in Passerines

	$d_{\text{n}}/d_{\text{s}}$	GC3	**τ**	Intron Number	Intron Length	Protein Length	Chromosome Size	Gene Density
*d*_s_	−0.04***	0.22***	0.10***	NS	−0.10***	NS	−0.10***	0.09***
$\text{ω}=d_{\text{n}}/d_{\text{s}}$	–	−0.17***	0.06***	−0.07***	−0.14***	−0.05***	0.07***	NS
GC3		–	0.19***	−0.13***	−0.19***	−0.12***	−0.35***	0.29***
τ			–	0.06***	0.11***	0.10***	NS	NS
Intron number				–	0.66***	0.79***	−0.10***	0.13***
Intron length					–	0.57***	0.04***	−0.11***
Protein length						–	−0.09***	0.11***
Chromosome size							–	−0.55***

Note.–*d*_s_ and $d_{\text{n}}/d_{\text{s}}$ were estimated using pairwise alignments between great tit and zebra finch. GC3, GC content at third codon position; τ, expression specificity; NS, not significant.

***P < 0.001.

**Table 3:** Partial Correlation Analyses Based on Kendall’s τ to Investigate the Effect of Variation in Various Covariates on the Correlation between the Two Variables of Interest

Case	Variables	Covariates	Kendall’s τ
1	ω, τ	GC3	0.047***
2	ω, τ	GC3, read depth	0.033***
3	ω, intron length	GC3, chromosome size	−0.031***
4	ω, intron number	GC3, chromosome size	−0.098***
5	ω, protein length	GC3, chromosome size	−0.028**
6	*d*_s_, chromosome size	GC3	−0.031***
7	ω, genomic location^a^	GC3, gene density, τ, intron number, protein length	0.02*^1^
8	ω, genomic location^b^	GC3, gene density, τ, intron number, protein length	0.023^NS^
9	ω,^c^ genomic location^a^	GC3, gene density, τ, intron number, protein length	0.075***
10	*p,*^d^ genomic location^a^	GC3, gene density, τ, intron number, protein length	0.047***
11	Δln *L*,^e^ genomic location^a^	GC3, gene density, τ, intron number, protein length	−0.13*^2^

Note.—NS, not significant.

^a^Outer parts of macrochromosomes and microchromosomes versus inner parts of macrochromosomes.

^b^Outer parts of macrochromosomes versus microchromosomes.

^c^ω at nonneutral sites (nearly neutral model M1a, fig. 3).

^d^Proportion of neutral sites (nearly neutral model M1a, fig. 3).

^e^Log-likelihood difference (model M7 vs. M8, test for positive selection, genes with *P* <1.0).

***P≤0.001.

**P≤0.01.

*^1^*P* = 0.032.

*^2^*P* = 0.027

^NS^*P* > 0.05.

The pairwise relationship between ω and several measures of gene compactness including the number of introns, total length of introns, and the length of the protein sequence are all significantly negative, in agreement with previous analysis of nonavian species (table 2, Larracuente et al. 2008; Choi and Hannenhalli 2013). Partial correlation analyses suggest (table 3, Cases 3–5) that none of these correlations were driven by of the apparent pairwise covariation between these gene features with GC3 (which covariates with ω) and chromosome size (which covariates with measure of gene compactness). The positive relationship between intron length and chromosome size is consistent with the fact that microchromosomes are more compact (International Chicken Genome Sequencing Consortium 2004; Nam and Ellegren 2012). We also found a negative correlation between ω and the proportion of sequences near exon–intron boundaries (Spearman ρ=−0.06 and *P* < 0.001), as reported in humans (Parmley et al. 2007).

Our analysis of the two-way alignments also unearths the following patterns, which have been reported in earlier studies of other bird genomes, confirming the high quality of our data and the generality of these patterns (Axelsson et al. 2005; Nam et al. 2010; Künstner et al. 2010; Balakrishnan et al. 2013; reviewed by Ellegren 2013). First, smaller chromosomes tend to have higher divergence at synonymous sites (table 2, *d*_s_ versus chromosome size). Interestingly, the correlation between *d*_s_ and chromosome size remains significantly negatively correlated after controlling for GC3 (table 3, Case 6), implying that the correlation was not entirely due to the positive relationship between GC3 and substitution rates (table 2; Webster et al. 2006). It is possible that smaller chromosomes may have higher mutation rates (Axelsson et al. 2005; Nam et al. 2010; Künstner et al. 2010). Secondly, there is a significant positive relationship between ω and chromosome size (table 2). This pattern is unlikely to be driven by the fact that smaller chromosomes tend to have higher gene density, as ω and gene density are not statistically correlated (table 2); nor does it seem probable that gene expression specificity, which is uncorrelated with chromosome size (table 2), has played a major role. In a later section, we will go beyond previous studies and investigate whether the dramatic variation in recombination rate among different genomic regions has contributed to this correlation through the process of HRI.

### Heterogeneity in ω between Genes Involved in Different Biological Functions

**Fig. 2.—:**
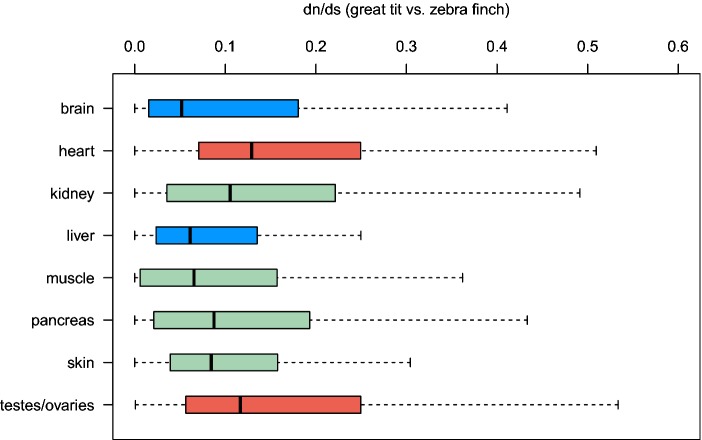
Boxplots of evolutionary rates for subsets of genes specifically expressed in certain tissues; boxes in blue and red denote significantly reduced and increased $d_{\text{n}}/d_{\text{s}}$ values, respectively. Whiskers were drawn as implemented in the R-function boxplot (see Materials and Methods).

We have shown that the rate of molecular evolution varies substantially in passerine birds across the genome. It is, however, unclear whether genes with issue-specific expression also have different ω’s. To answer this question, we extracted tissue-specific genes for the eight tissues included in the transcriptome sequencing, and compared their ω values (fig. 2). The median ω value for genes specifically expressed in the brain is 0.051, which is significantly lower than the genome-wide median of 0.1 (Mann–Whitney *U* test [MWU], *P* = 0.005) and the median value of other tissue-specific genes (MWU, *P* = 0.021). This could be explained by the theory put forward by Drummond and Wilke (2008, see Introduction). In contrast, testis/ovary-specific genes have significantly increased evolutionary rates when compared with other tissue-specific genes (MWU, *P* = 0.027), consistent with the intra- and interspecific arms race theory. Interestingly, genes specifically expressed in the heart had the highest median ω = 0.129, which is comparable to that of the testis/ovary-specific genes (MWU, *P* = 0.15). This is different from humans whose heart-specific genes have significantly lower median ω (0.07) than testis-specific genes (0.103) (Winter et al. 2004). The cause of this difference is unclear and warrants investigation in future.

We further tested whether genes with very low (smaller than the 10th percentile, likely to contain many genes under strong selective constraints) or very high (larger than the 90th percentile, likely to include genes either under relaxed constraints or fast-evolving genes driven by recurring episodes of positive selection) ω values are enriched for particular GO categories. Seven GO terms have significantly more low-ω genes than expected by random chance (Fisher’s exact test with Bonferroni correction *P* < 0.01, supplementary table S2, Supplementary Material online); these include genes involved in core cellular functions such as ribosomal complexes or metabolic regulation. Genes associated with at least one of these seven GO terms tend to have lower expression specificity (MWU, *P* < 0.001), which is expected for housekeeping genes (Zhang and Li 2004). For genes with elevated ω, we did not observe significant over- or underrepresentation in any GO terms. However, in light of the potential problems of GO-based analysis (Pavlidis et al. 2012), these results should be regarded as exploratory.

### Evidence of More Effective Natural Selection in Regions with Frequent Recombination

**Fig. 3.—:**
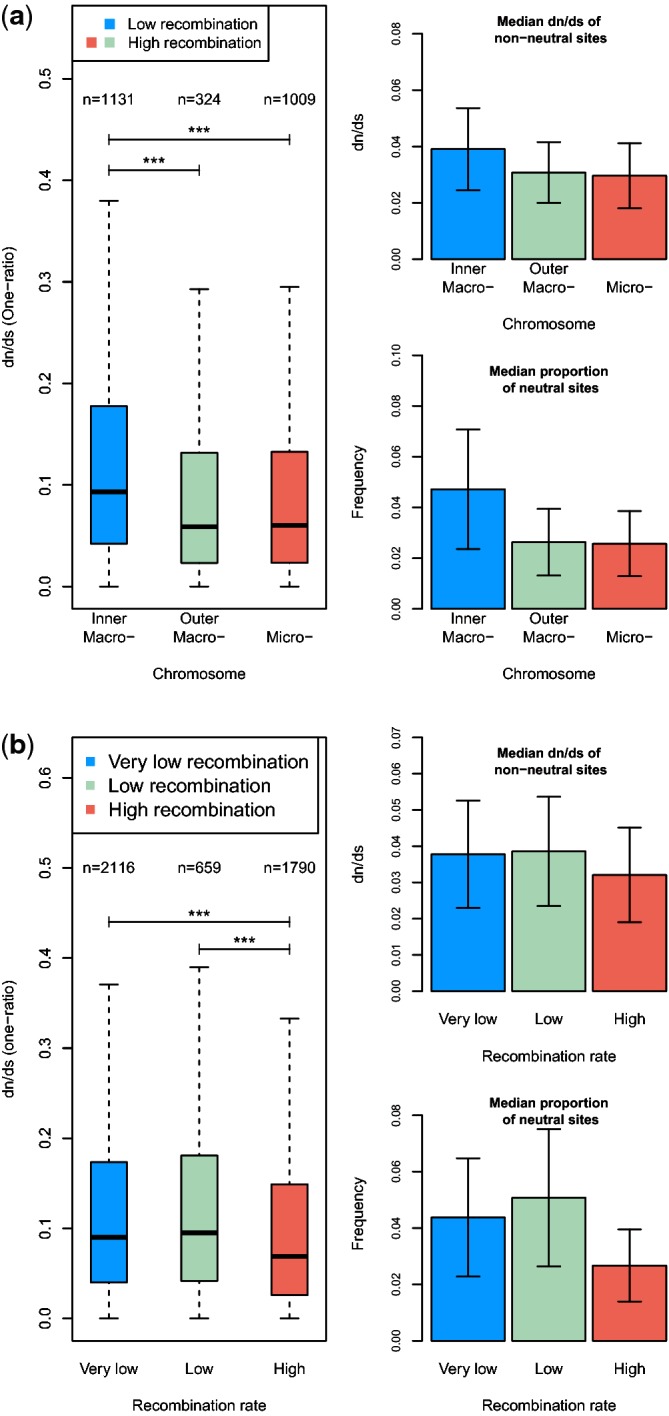
Box plots of evolutionary rates for subsets of genes according to their (*a*) chromosomal positions and (*b*) recombination rate estimates. Whiskers are drawn as implemented in the R-function box plot (see Materials and Methods). The bar plots show the proportion of sites estimated to be evolving under neutrality and the median $d_{\text{n}}/d_{\text{s}}$ value for sites inferred to be evolving under purifying selection by the nearly neutral (model M1a) in PAML. Error bars indicate median absolute deviations (MDA). ${}^{***}P\leq 0.001$ under the MWU.

We first tested whether variation in local recombination rates contributes to the covariation between ω and chromosome size. Given the pronounced telomere effect observed in macrochromosomes and the substantial differences in average recombination rate between macro- and microchromosomes, we defined three sets of genes: 1) genes in central parts of macrochromosomes (low recombination frequency); 2) genes located near ends of macrochromosomes (i.e., subtelomeric regions, highly recombining); and 3) genes in microchromosomes (highly recombining). The ends of macrochromosomes and microchromosomes are often referred to as recombination jungles, and central parts of macrochromosomes as recombination deserts (e.g., Backström et al. 2010). Using the MWU, we found that the genes in recombinations deserts have significantly higher ω values than the other two sets (fig. 3*a*; *P* = 0.002). Because the MWU cannot control for the effects of covariates, we used a partial correlation method to test whether ω was positively correlated with a genomic location variable, which took the value of 0 or 1 for genes located in recombination jungles or deserts, respectively. We chose GC3, gene density, expression specificity, intron number, and protein length as covariates but did not consider chromosome size. This is because recombination jungles and deserts were defined mainly in light of the fact that microchromosomes have, on average, much higher recombination rates per base pair than macrochromosomes, but this relationship would disappear if chromosome size was held constant. After controlling for covariates, ω was found to be significantly lower in recombination jungles (table 3, Case 7). Interestingly, ω is not statistically different between the two high-recombination sets (fig. 3*a*; MWU, *P* = 0.13), and this remains the case when covariates were controlled for (table 3, Case 8).

To check whether the above results are robust to how regions with frequent and infrequent recombination are defined, we estimated local recombination rates by comparing the zebra finch genetic map with its reference genome, and defined high-, low-, and very low-recombination regions (Backström et al. 2010; Warren et al. 2010, see Materials and Methods). Genes in high-recombination regions show a reduction regarding their median ω value when compared with either very low- or low-recombination regions (MWU, *P* = 0.001 and *P* = 0.002, respectively), which is consistent with the pattern found above. No significant difference in ω was found between regions with low and very low recombination rate estimates (MWU, *P* = 0.2). Given the fact that the majority of the genes estimated to have low- and very low-recombination rates were not located at the ends of macrochromosomes or microchromosomes (2,607 out of 2,767, ≈94%), these results suggest that ω and local recombination rates are negatively correlated and that the overall difference in ω between macro- and microchromosomes (e.g., ω versus chromosome size in table 2) may be in part driven by high ω in low-recombination regions of macrochromosomes (i.e., recombination deserts).

Next we asked whether purifying selection is less effective in regions with infrequent recombination, as predicted by the HRI theory (see Introduction). To increase statistical power, we used three-way alignments including orthologous genes from the chicken genome in this analysis. We also excluded genes showing evidence of positive selection according to a “site model” implemented in PAML (at an FDR level of 10%; see Materials and Methods). We used PAML to estimate, for each locus, 1) the proportion of neutrally evolving sites and 2) ω at sites that are under purifying selection. As above, genes were assigned to three different groups according to their recombination rates. Compared with genes found in the recombination jungles, genes located in recombination deserts have a significantly higher proportion of neutral sites (fig. 3*a*; MWU, *P* = 0.001 and *P* < 0.001 for comparisons with ends of macrochromosomes and microchromosomes, respectively) and significantly higher ω at sites under purifying selection (fig. 3*a*; MWU, *P* = 0.002 and *P* < 0.001). After controlling for the covariates mentioned above, ω for nonneutral sites and the proportion of neutral sites were found to be significantly lower in recombination jungles (table 3, Cases 9 and 10)

Similar patterns can be seen when genes in either low- or very low-recombination regions were compared with those in high-recombination regions (fig. 3*b*; *P* < 0.005 for all comparisons). These patterns are consistent with relaxed selective constraints in regions where recombination is infrequent. Interestingly, microchromosomes and ends of macrochromosomes have very similar median values of the proportion of neutral substitutions and ω for nonneutral substitutions (fig. 3*a*; MWU, *P* > 0.1). Similarly, no statistically significant difference was found between low- and very low-recombination regions (fig. 3*b*; MWU, *P* > 0.1).

Finally, we examine whether positive selection is also more efficient in regions with higher recombination frequencies. Support for positive selection was determined by using a site model implemented in PAML (see Materials and Methods). Among the 1,333 genes in recombination jungles, ten show evidence for positive selection, which is significantly more frequent than genes in recombination deserts where 2 out of 1,131 genes have experienced positive selection (table 4; with an FDR level of 10%; G test, *P* = 0.03). Because the G test cannot take into account covariates, we carried out the following analyses. If high local recombination rates facilitate the fixation of beneficial variants, then the M8 model should, on average, fit the data better than the M7 model, and therefore difference in ln likelihood between the two models (ΔlnL) should be larger in high-recombination regions. Indeed, controlling for covariates, model M8, which includes positive selection, fitted the data from high-recombination regions better (table 3, Case 11).

**Table 4:** Location of Genes with Evidence for Positive Selection

FDR	Recombination Region	Positively Selected	Not Positively Selected	*P *(G Test)
10%	High	8	1,782	
	Low	0	659	0.02
10%	Jungle^a^	10	1,323	
	Desert^b^	2	1,129	0.03

Note.—Genes were classified according to their genomic locations (recombination jungles and deserts) or their estimated local recombination rate (high- and low/very low recombination, see Materials and Methods). Comparisons were conducted between genes located in different recombinational environments using G tests.

^a^Outer parts of macrochromosomes and microchromosomes.

^b^Inner parts of macrochromosomes.

A similar enrichment of positively selected loci is also found in high-recombination regions, relative to low recombination regions (table 4; G test, P=0.02). These results (as well as those presented earlier in this section) are robust to different definitions of regions with different recombination frequencies and different FDR thresholds (supplementary table S3, Supplementary Material online) and the use of a different combination of sequence aligner and alignment processing algorithm (PRANK and GUIDANCE; see Materials and Methods; supplementary table S4, Supplementary Material online) or a more robust but less powerful model comparison to detect positive selection (PAML; M1a vs. M2a, supplementary table S5, Supplementary Material online). Thus, in agreement with the HRI theory, elevated local recombination reduces interference between linked selected sites and facilitates both the spread of beneficial mutations and the removal of deleterious mutations.

## Discussion

In this study, we used a multitissue transcriptome data set in great tits, together with the reference genomes of the zebra finch and the chicken, to study patterns of molecular evolution along the two passerine lineages. By contrasting patterns of sequence divergence between genes in high- and low-recombination regions of the genome and by analyzing genes with and without evidence of positive selection separately, we obtained evidence that the efficacy of both positive and negative selection is higher in regions with more frequent recombination, as predicted by the HRI theory (fig. 3 and table 4). We also showed that more compact genes with fewer introns, shorter introns, and shorter proteins tend to evolve faster (table 2) and that genes with a larger proportion of exon–intron boundaries have lower ω. The latter two results have not previously been examined in birds (Parmley et al. 2007; Choi and Hannenhalli 2013). These analyses demonstrate that transcriptome sequencing is a powerful way to address fundamental questions about genome evolution in organisms such as great tits where genomic resources are relatively limited.

### Gene Expression Pattern as a Major Predictor of Protein Evolution

Gene expression pattern can be viewed as encompassing both expression level and tissue specificity of expression. Although it is well known that gene expression level is a key predictor of ω (reviewed by Pál et al. 2006 and Choi and Hannenhalli 2013), this factor was not considered in this study because our cDNA libraries were normalized prior to sequencing (Santure et al. 2011), which means that read depth is probably an unreliable measure of gene expression level. Nonetheless, when read depth was used as a proxy of expression level, we did find a significant negative correlation between ω and expression level (Spearman’s ρ = −0.06, *P* < 0.001), which is in the same direction as reported previously in many organisms (reviewed by Pál et al. 2006 and Choi and Hannenhalli 2013).

Our finding of a positive correlation between tissue specificity in expression (τ) and ω should be conservative in the presence of library normalization, as this procedure suppresses differences in expression level, and is therefore expected to homogenize differences between tissues (Ekblom et al. 2012). This may explain why the pairwise correlation between τ and ω reported in table 2 is somewhat weaker than those presented in previous studies (e.g., 0.3 in *Drosophila*, 0.24 in mice, 0.12 in humans; Larracuente et al. 2008; Park et al. 2012). Evidence for this homogenization is provided by an analysis conducted on contigs assembled from at least 50 reads (combined across all tissues), the correlation increased to 0.11, which may reflect that estimates of tissue specificity were more accurate when more reads were available. A possible biological explanation of the relationship between τ and ω is increased pleiotropy: Proteins that are expressed in many tissues may tend to have more interacting partners, which lead to more constraints on the function and/or structure of the protein, and a corresponding reduction in evolutionary rate (Pál et al. 2006). There is evidence that τ and expression level are highly correlated in some species (Lercher et al. 2002; Subramanian and Kumar 2004) including birds (Ekblom et al. 2010). In mammals, tissue specificity seems to explain more of the variation in rates of protein evolution than expression level (Pál et al. 2006). It is possible that the positive correlation we observe here may be in part driven by variation in gene expression level. Better data are needed to establish the relative importance of the breadth and level of expression in determining evolutionary rates in passerines.

Although genes with high tissue specificity in expression tend to evolve faster as a whole, the distribution of ω is highly heterogeneous among tissues (fig. 2). Specifically, genes expressed mainly in the brain have, on average, the lowest ω, which is consistent with findings in metazoans (Axelsson et al. 2008; Drummond and Wilke 2008), and is probably due to the fact that neuronal tissues are particularly sensitive to the damaging effects of mistranslation-induced protein misfolding. Therefore, these genes are under strong selective constraints because of the rarity of well-adapted sequences with high translational accuracy and robustness (Drummond and Wilke 2008). On the other hand, genes expressed in testis/ovary have accelerated rates of molecular evolution. This pattern, which has been observed in other organisms such as humans (Nielsen et al. 2005) and *Drosophila* (Zhang et al. 2004), can potentially be caused, either individually or in some combination, by sperm competition, sexual selection, and sexual conflict (Swanson and Vacquier 2002), all of which are common in birds (Birkhead and Moller 1992). Additional research is needed to clarify the relative importance of these factors in birds.

### HRI as a Determinant of Protein Evolution in Birds

Our analysis of patterns of protein evolution in the two passerine lineages suggests that HRI is likely to have played an important role in determining variation in ω. In particular, regions with reduced recombination tend to be more prone to the accumulation of slightly deleterious substitutions, whereas the fixation of beneficial mutations is more likely to take place in high-recombination regions (fig. 3 and table 4). These results are insensitive to different definitions of high- and low-recombination regions and FDR cutoffs. Intrachromosomal rearrangements, which have occurred between the three bird species considered (reviewed by Ellegren 2010, 2014; see also van Oers et al. 2014), should not make the test counterconservative. This is because, for macrochromosomes, shuffling genes between the ends and the central parts is expected to homogenize differences in recombination frequency, whereas for microchromosomes, genetic maps in all species studied to date suggest recombination rates are roughly constant along the length of the chromosome (Backström et al. 2010; Stapley et al. 2010; van Oers et al. 2014).

**Fig. 4.—:**
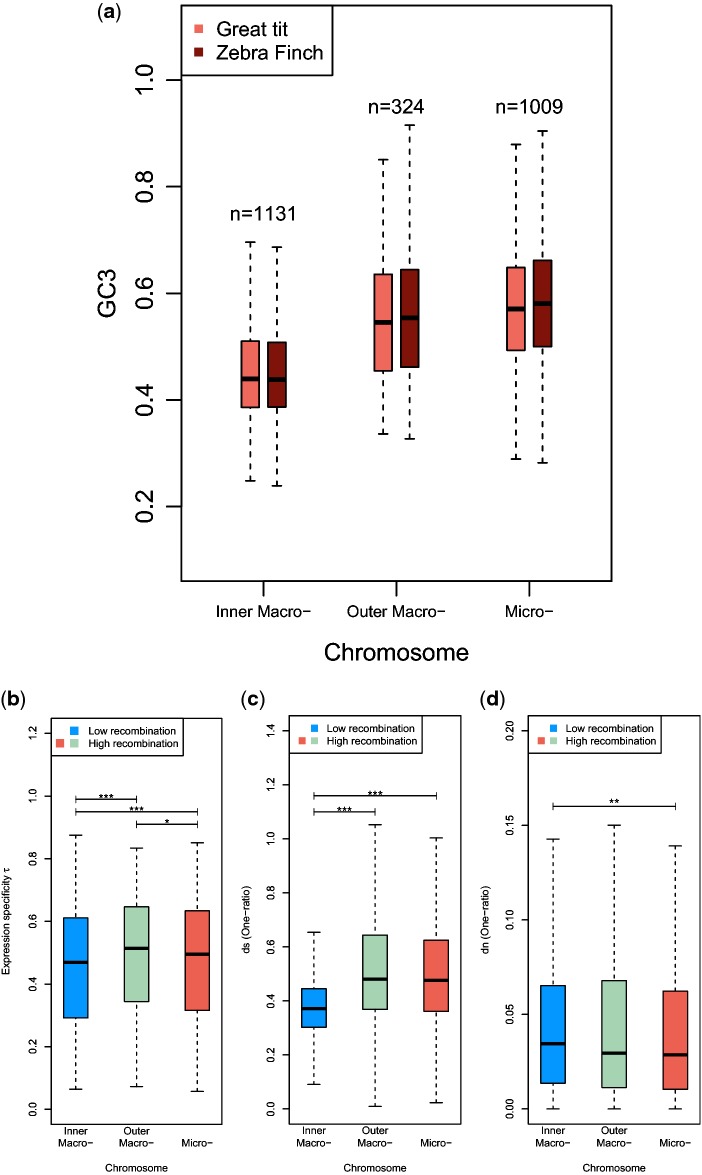
Box plots of (*a*) GC content at 3rd positions (GC3), (*b*) expression specificity τ, (*c*) *d*_s_, and (*d*) *d*_n_ for subsets of genes according to their chromosomal positions. Whiskers are drawn as implemented in the R-function box plot (see Materials and Methods). ${}^{***}P\leq 0.001$, ${}^{**}P\leq 0.01$, ${}^{*}P\leq 0.05$.

There is evidence that GC content is not at statistical equilibrium in multiple avian lineages and that GC-biased gene conversion (gBGC) may have contributed to this (Webster et al. 2006; Nabholz et al. 2011; Mugal et al. 2013). However, our results should also be robust to variation in GC content and the action gBGC, which can upwardly bias estimates of ω and lead to false detection of targets of positive selection (reviewed by Duret and Galtier 2009). First, we used the site models in PAML, which analyzed substitution patterns over the entire phylogenetic tree in the search of positively selected genes. A recent analysis has shown that results produced by this approach are unlikely to be affected by gBGC (Ratnakumar et al. 2010). Second, if substitutions of slightly deleterious mutations were driven by gBGC (Galtier et al. 2009), we expect this effect to be stronger in regions with higher recombination rates and GC content, which are often used as proxies of the intensity of gBGC (Duret and Galtier 2009). Contrary to this prediction, in regions with reduced recombination, where ω is higher, GC content is lower (figs. 3 and 4*a*), and evidence of relaxed constraints on nonsynonymous sites also comes from these regions (fig. 4*d*).

There is little evidence that the systematic difference in ω displayed in figure 3 is driven by a similar difference in tissue specificity in gene expression (Case 7 in table 3). As shown in figure 4*b*, regions with frequent recombination actually have significantly higher τ than those with reduced recombination. A similar relationship between τ and recombination has been observed in humans (Necsulea et al. 2009) and *Drosophila* (Weber and Hurst 2011). We currently do not have data to ascertain whether gene expression level differ between these genomic regions. However, as mentioned above, if we were to assume that there is a strong negative correlation between τ and expression level (Lercher et al. 2002; Subramanian and Kumar 2004; Ekblom et al. 2010), then expression level would be lower in regions with frequent recombination and therefore would not be the main driver of the difference in ω.

Because ω is defined as the ratio of *d*_n_ to *d*_s_, it may be inflated when *d*_s_ is unusually small either due to random chance or selective constraints on synonymous sites, resulting in false detection of positive selection. A recent analysis based on three-species alignments of chicken, turkey, and zebra finch has reported evidence that synonymous sites may be under significant constraints (Künstner et al. 2011). However, our results, which are based on comparisons of the number of selected genes detected by PAML between different classes of genes, should be robust. First, Künstner et al. (2011) found no evidence of regional variation in selective pressure on synonymous sites and suggested that difference in *d*_s_ between regions reflect variation in mutation rate. Second, the median *d*_s_ values of the positively selected genes (at an FDR level of 10%) and the other genes were 0.39 and 0.4, respectively, which were not significantly different (MWU, *P* = 0.25). Third, as shown in figure 4*c*, regions with high recombination rates tend to have much higher *d*_s_ than those with infrequent recombination. These three observations suggest that genes in high-recombination regions should not be more prone to false detection of positive selection. Hence, it seems unlikely that our results can be explained by selection at synonymous sites.

Our results therefore extend previous analyses of the negative correlation between recombination rate and ω in birds (Axelsson et al. 2005; Nam et al. 2010; Künstner et al. 2010; Balakrishnan et al. 2013) by showing that 1) higher ω in low-recombination regions is due to relaxed purifying selection rather than enrichment of fast-evolving genes driven by positive selection, 2) that frequent recombination facilitates the incorporation of new beneficial mutations, and 3) that microchromosomes and ends of macrochromosomes show very similar patterns of protein evolution as a consequence of frequent recombination. The positive relationship between the efficacy of selection and recombination rate appears to be consistent with the observation that diversity at putatively neutral sites (a proxy of local *N*_e_; Charlesworth 2009) increases with local recombination rates in several birds (reviewed by Ellegren 2013). However, further research is needed to test whether evidence of HRI indeed exists both within and between species.

There are differences between the passerines and other organisms in terms of observations related to HRI. For instance, in *Drosophila*, differences in ω are most visible between regions that lack recombination (e.g., the fourth chromosome) and those where crossing-over occurs, whereas within the crossover regions, little difference in ω was found between regions with high, intermediate, and low crossover frequencies (Haddrill et al. 2007; Larracuente et al. 2008). However, in our case, ω appears to be more variable within the crossover regions, with regions with low, but nonzero, recombination rates having significantly higher ω than high-recombination regions (fig. 3*b*). The enrichment of targets of positive selection in high-recombination regions also contrasts with the lack of such enrichment in humans (Bullaughey et al. 2008). It is possible that the highly variable recombinational landscape in birds has made the effects of HRI more obvious across genomic regions. It is also possible that selection is more effective in birds than in humans, which may make it easier to detect HRI in the former (Bullaughey et al. 2008). Evidence of more effective selection in birds than in humans can be seen from the observation that a higher proportion of nonsynonymous substitutions in birds may be driven to fixation by positive selection than in humans (Eyre-Walker 2006; Axelsson and Ellegren 2009) and that birds have lower average ω (≈0.15) than humans (≈0.33; Zhang et al. 2013; supplementary table S1, Supplementary Material online). However, as pointed out by Cutter and Payseur (2013) (see also Connallon and Knowles 2007; Webster and Hurst 2012), predictions of HRI depend in a complicated way on parameters such as recombination rate, distribution of fitness effects of new mutations, and effective population size. More analysis is necessary to characterize these parameters in birds, which shall in turn facilitate comparisons with other species.

## Supplementary Material

Supplementary tables S1–S4 and figures S1–S8 are available at *Genome Biology and Evolution *online (http://www.gbe.oxfordjournals.org/).

Supplementary Data
