# Supplementary material for: Highly Variable Recombinational Landscape Modulates Efficacy of Natural Selection in Birds
Source: Genome Biol Evol. 2014 Jul 24;6(8):2061–75. doi: 10.1093/gbe/evu157 (PMC4231635; doi:10.1093/gbe/evu157)
Supplement: Supplementary Data [file supp_6_8_2061__index.html]

Highly variable recombinational landscape modulates efficacy of natural selection in birds — Highly Variable Recombinational Landscape Modulates Efficacy of Natural Selection in Birds — Supplementary Data 

# Highly Variable Recombinational Landscape Modulates Efficacy of Natural Selection in Birds

## Supplementary Data

files

**Files in this Data Supplement:**

- Supplementary Data - pdf file
