# Supplementary material for: Highly Variable Recombinational Landscape Modulates Efficacy of Natural Selection in Birds
Source: Genome Biol Evol. 2014 Jul 24;6(8):2061–75. doi: 10.1093/gbe/evu157 (PMC4231635; doi:10.1093/gbe/evu157)
Supplement: Supplementary Data [file supp_evu157_gbe-140421.pdf]

Supplementary information

Comparison	$\overline{d_s}$	$\overline{d_n}/\overline{d_s}$
D. melanogaster vs. *D. simulans*	0.118	0.12
Mouse vs. rat	0.174	0.14
**Great tit vs. zebra finch**	**0.111**	**0.16**
A. thaliana vs. *A. lyrata*	0.139	0.21
Human vs. chimpanzee	0.029	0.33

Table S1: Mean estimates of d_s and d_n/d_s. Values except for the great tit vs zebra finch comparison are from Zhang et al. (2013).

GO term	Description	Observation	Expectation	P-value (bonferroni)
GO:0032991	macromolecular complex	162/482	981/4652	1.09E-06
GO:0030529	ribonucleoprotein complex	38/482	148/4652	0.000215
GO:0043234	protein complex	130/482	843/4652	0.00137
GO:0010468	regulation of gene expression	93/482	548/4652	0.00169
GO:0005488	binding	332/482	2724/4652	0.00369
GO:0044391	ribosomal subunit	14/482	33/4652	0.00662
GO:0019222	regulation of metabolic process	129/482	859/4652	0.00823

Table S2: GO terms that are enriched among genes with low d_n/d_s values.

FDR	Recombination region	Positively Selected	Not positively selected	P-value (G-test)
20%	High	17	1773	
	Low	1	658	0.01
30%	High	20	1770	
	Low	1	658	0.01
40%	High	25	1765	
	Low	4	655	0.08
50%	High	31	1759	
	Low	6	653	0.11
20%	Jungle [a]	15	1318	
	Desert [b]	6	1125	0.1
30%	Jungle [a]	21	1312	
	Desert [b]	8	1123	0.04
40%	Jungle [a]	27	1306	
	Desert [b]	12	1119	0.05
50%	Jungle [a]	33	1300	
	Desert [b]	16	1115	0.05
10%	High	8	1782	
	Low+very low	6	2769	0.17
20%	High	17	1773	
	Low+very low	12	2763	0.03
30%	High	20	1770	
	Low+very low	17	2758	0.06
40%	High	25	1765	
	Low+very low	26	2749	0.15
50%	High	31	1759	
	Low+very low	31	2744	0.08
10%	Jungle [c]	15	2630	
	Desert [d]	2	1706	0.01
20%	Jungle [c]	22	2623	
	Desert [d]	8	1700	0.14
30%	Jungle [c]	29	2616	
	Desert [d]	10	1698	0.07
40%	Jungle [c]	39	2606	
	Desert [d]	15	1693	0.07
50%	Jungle [c]	49	2596	
	Desert [d]	20	1688	0.07

[a] outer parts (3 Mb closest to the tip) of macrochromosomes and microchromosomes

[b] Inner 25% of macrochromosomes

[c] outer parts (15 Mb closest to the tip) of macrochromosomes and microchromosomes

[d] Inner 50% of macrochromosomes

Table S3: Location of genes with evidence for positive selection at different FDRs. Comparisons are conducted between recombination jungles and recombination deserts using G-tests. Recombination regions are classified according to their recombination rate estimates (low versus high recombination) and chromosomal positions (recombination jungle versus desert). FDR; False discovery rate

FDR	Recombination region	Positively Selected	Not positively selected	P-value (G-test)
10%	High	6	1784	
	Low	0	659	0.05
10%	Jungle [a]	7	1326	
	Desert [b]	0	1131	0.003

[a] Outer parts of macrochromosomes and microchromosomes

[b] Inner parts of macrochromosomes

Table S4: Location of genes with evidence for positive selection using a different alignment strategy (PRANK and GUIDANCE). Comparisons were conducted between recombination jungles and recombination deserts using G-tests. Recombination regions were classified according to their recombination rate estimates (low versus high recombination) and chromosomal positions (recombination jungle versus desert). FDR; False discovery rate

FDR	Recombination region	Positively Selected	Not positively selected	P-value (G-test)
10%	High	7	1783	
	Low	0	659	0.04
10%	Jungle [a]	9	1324	
	Desert [b]	1	1130	0.01

[a] Outer parts of macrochromosomes and microchromosomes

[b] Inner parts of macrochromosomes

Table S5: Location of genes with evidence for positive selection using model comparison M1a-M2a from PAML (Yang, 2007). Comparisons were conducted between recombination jungles and recombination deserts using G-tests. Recombination regions were classified according to their recombination rate estimates (low versus high recombination) and chromosomal positions (recombination jungle versus desert). FDR; False discovery rate

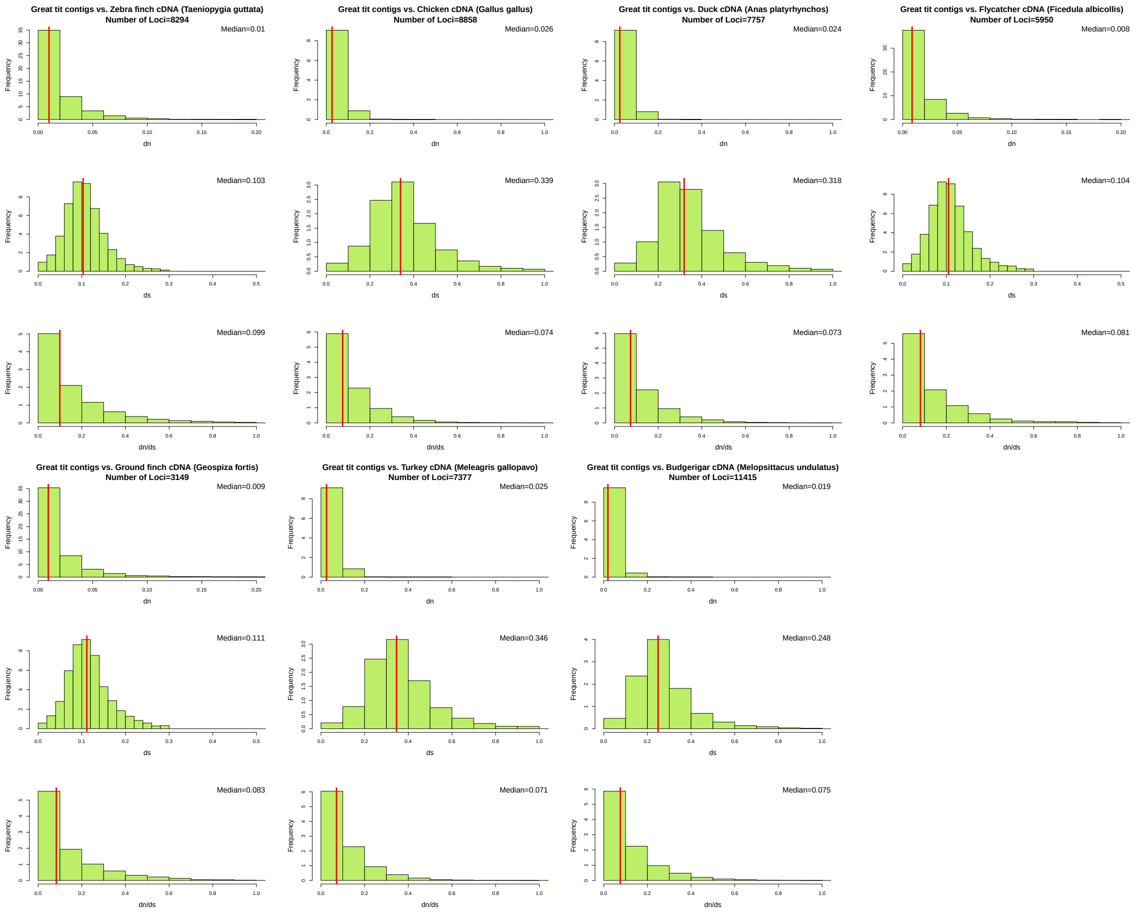

Figure S1: Distribution of dn, ds and d_n/d_s derived from great tit contigs mapped to the chicken and zebra finch genome, respectively. Median values are indicated by solid red lines.
